# Sensors in Agriculture and Forestry

**DOI:** 10.3390/s130912132

**Published:** 2013-09-10

**Authors:** Gonzalo Pajares, Andrea Peruzzi, Pablo Gonzalez-de-Santos

**Affiliations:** 1 Department of Software Engineering and Artificial Intelligence, Faculty of Informatics, University Complutense of Madrid, 28040 Madrid, Spain; E-Mail: pajares@ucm.es; Tel.: +34-1-394-7546; Fax: +34-1-394-7547; 2 Department of Agriculture, Food and Environment, University of Pisa, Via S. Michele degli Scalzi 2, 56124 Pisa, Italy; E-Mail: andrea.peruzzi@unipi.it; Tel.: +39-050-221-8942; Fax: +39-050-221-8966; 3 Centre for Automation and Robotics (UPM-CSIC), Arganda del Rey 28500, Madrid, Spain; E-Mail: pablo.gonzalez@car.upm-csic.es; Tel.: +34-1-871-1900; Fax: +34-1-871-7050

Agriculture and Forestry are two broad and promising areas demanding technological solutions with the aim of increasing production or accurate inventories for sustainability while the environmental impact is minimized by reducing the application of agro-chemicals and increasing the use of environmental friendly agronomical practices. In addition, the immediate consequence of this “trend” is the reduction of production costs.

Sensors-based technologies provide appropriate tools to achieve the above mentioned goals. The explosive technological advances and development in recent years enormously facilitates the attainment of these objectives removing many barriers for their implementation, including the reservations expressed by the farmers themselves. Precision Agriculture is an emerging area where sensor-based technologies play an important role.

Farmers, researchers and technical manufacturers, all together are joining their efforts to find efficient solutions, improvements in production and reductions in costs. The dissemination of results through scientific publications and international events, among which Sensors is a clear example, contributes significantly to this effort. This is the second special issue published in Sensors for the same topic (Agriculture and Forestry) with important contributions; some of them coming from the First International Conference on Robotics and associated High-technologies and Equipment (www.rhea-conference.eu/2012), emphasizing how much sensitivity and coverage Sensors offer for technological advancement in these areas.

There are still many challenges and problems to be solved or improved. Therefore, new special issues are foreseen to be launched in the future, including the one in conjunction with the above mentioned conference on its second edition (http://www.rhea-conference.eu/2014/).

After the call for papers we received tens of manuscripts, 41 high quality of which were finally selected after a peer review process conducted by prestigious scientists with a high expertise in the different covered topics.

Sensors are selected or designed according to the problems to be solved or needs identified by farmers. The papers published on this special issue are here grouped based on the application they address, specifying the type of sensor or sensors used and the methods developed or used for such purpose.

## Soil Analysis and Characteristics

Soil is an essential element in Agriculture as it plays a fundamental role for crop development, yield and products quality, both for its intrinsic properties and the affectation of external factors. In [1] a spectroradiometer is the sensor used for hyperspectral analysis of soil contents (nitrogen, carbon and organic matter) based on regression trees. Soil salinity assessment of sandy mineral and bulk electrical conductivity is addressed in [2] based on frequency-domain reflectometry. The salinity index is defined as partial derivative of the soil bulk electrical conductivity with respect to the real part of the soil complex dielectric permittivity. Soil moisture, temperature and electrical conductivity are analyzed in [3] by applying also time domain reflectometry (TDR). The device is designed with eight channels of TDR, two-rod probes and a General Packet Radio Service modem for collecting data. In [4] the soil detachment by water drop impact is analyzed at laboratory level by using a light microscope. Image analysis, based on image processing methods of splashed particles, is the technique applied. In [5] soil water content and salinity is monitored through a low-cost capacitance resistance by direct measurements. In [6] an automatic resistivity profiler together with a GPS system are used in vineyard to assess the spatial variability patterns of vegetative growth and yield based on the analysis of soil electrical resistivity and ancillary topographic attributes (elevation, slope). In many agricultural practices, the soil is affected and compacted by the transit of agricultural machinery, causing a appreciable decrease in crop yield; under this point of view, the effects of tractor field usage is studied in [7] with the design of a device equipped with an electrical penetrometer, which measures the resistance of soils before and after the vehicle passes. Soil cutting resistance for site specific tillage is studied in [8], using a tool constituted by four blades equipped with load sensors.

## Yield

Yield estimation or impacts on yield are topics of great interest. Vegetative growth and yield, based on soil electrical resistivity and ancillary topographic, are studied in [6]. An analysis of the effects on yield caused by a decrease of soil compaction is reported in [7]. A study of sugarcane yield estimation carried out by means of a GreenSeeker^®^ device and based on normalized difference vegetative indices is addressed in [9]. In [10] grapevine yield and leaf area estimation are studied with a RGB camera and natural illumination by applying image morphological operations and supervision classification approaches.

## Detection and Classification of Crops, Weeds, Fruits

Detection of relevant elements in agriculture or forestry for automatic processes is one of the essential tasks. In [11,12] red peaches in orchard images are detected through a RGB camera based on linear color models through a distance-based classification approach. In [12] a real-time system based on a processor with ARM architecture and Look-up-tables on the RGB color model are used for speed-up the fruit picking. The combination of an ultrasonic sensor and a camera are used for weed detection in cereal crop in [13]; the ultrasonic identifies height of plants and the camera determines the weed and crop coverage. In [14] a low cost smart camera is adapted with selected filters to pass red and near-infrared spectral bands and the Normalized Difference Vegetation Index (NDVI) is obtained for plant detection. Different issues related with crop and weeds detection accuracy is addressed in [15], where a camera-based sensor is geometrically arranged. The “vignetting” effect is studied, according to the use of ultra-violet and infra-red cut filters, and also the exposure time adjusted for image acquisition in outdoor environments. The development of a color vision-based system is reported in [16] for ripeness classification of oil palm fresh fruit bunch applying Artificial Neural Networks (ANN) and Principal Component Analysis (PCA). Fruit grading is addressed in [17] on the basis of a mobile sensing platform mounted on a glove that integrates several sensors, such as touch pressure, imaging, inertial measurements, localization and a Radio Frequency Identification (RFID) reader.

## Weed Control

Once weeds have been detected, the next step is their complete control. In [18] is reported the design and realization of a machine able to perform weed control both in inter-row and intra-row space. The implement actuates in the same time inter-row cultivation and intra-row band spraying by means of an electro-hydraulic side-shift frame using RTK-GPS. In [19] the reduction of weed competition in wheat and barley is controlled using a harrow equipped with bi-spectral cameras, which detect crop leaf cover, weed cover and soil density.

## Taste and Odor Detection

Some tastes and odors are of special interest in some agriculture and forestry applications. In [20] is described an electronic tongue realized and used to monitor the presence of glyphosate (a non-selective systemic herbicide) and discriminate potential interferents, using PCA as the method to analyze the redox processes produced. In [21] a review of diverse applications based on electronic-nose technologies is presented. Many factors, including humidity, available moisture, light, temperature, soil conditions, fertilization, insects and plant diseases may affect the release of volatile organic compounds from agricultural plants or trees in forestry, thus altering the normal odor. Some applications reported in [21] combine both electronic noses and electronic tongues.

## Positioning, Navigation and Safety

In automatic applications involving autonomous vehicles (tractors) this issue is of vital relevance. Global and local positions are required. The first can be established through a GPS system and the second based on crop rows detection. During navigation, safety to avoid obstacles or animals is a topic to be addressed. The Inertial Measurement Unit (IMU) is the key sensor used in [22] for positioning and navigation for land vehicles, where a fast orthogonal search modeling technique is applied. A LASER is the sensor used for auto-guidance vehicles for greenhouse operations in [23]. Laser emitters are to be detected and followed by the vehicle. Regarding the safety, in [24] a procedure for animal's detection is designed. It is based on Forward Looking Infrared (FLIR) cameras that detect gradients of temperature, and then image processing techniques based on thresholding are applied.

## Seeds, Seedling, Breeding, Growing and State of Health

The control of seeds and seedling to gain effectiveness and efficiency in the germination, emergence and determination of the different growth stages of crops represents a major challenge for agriculture. Some of them are also valid for commercialization purposes. Non-destructive control breeding and growing are also of interest in agriculture. In [25] maize seeds are discriminated through hyperspectral imaging techniques based on visible and infrared information, where the analysis of textures provides this information. Several classification-based techniques are applied with this purpose, such as PCA, Support Vector Machines or ANN. In [26] thermal imaging, based on infrared sensors, is applied to measure lettuce seed viability; regression analysis based on time-dependent thermal decay characterization is the technique applied. Moisture detection in rice grains based on microwave measurement techniques with a slim and small open-coded coaxial probe is the application described in [27]. In [28] a machine vision system based on image processing is described. In this case a vegetation index is applied for segmentation and shape feature generation and plant silhouettes are used as features and inputs for the classifiers (K-nearest neighbor, Bayes, Support Vector Machines). A multisensory platform for non-destructive field-based phenotyping (plant moisture content, cereal tiller density or biomass yield among others) in plant breeding is described in [29]. Various optical sensors like light curtain imaging, 3D Time-of-Flight cameras, laser distance sensors, hyperspectral imaging as well as color imaging are integrated into the platform. In [30] a configurable growth chamber with a computer vision system and controlled illumination is designed with the aim of study the circadian rhythm in plants, where form and shape variations at defined time intervals can be explored. The nitrogen uptake in winter wheat determines the posterior growth state for plants. In [31] a three sensor system is designed to obtain the canopy spectral reflectance for monitoring the above-ground plant nitrogen uptake. Chlorophyll function in photosynthesis is an important issue in plants. Chlorophyll fluorescence can be defined as the red and far-red light emitted by photosynthetic tissue when it is excited by a light source. This is an important phenomenon which permits to obtain important information about the state of health of a photosynthetic sample. In [32] a revision is presented about the current state of the art regarding the design of chlorophyll fluorescence sensing systems.

## Microorganisms and Pest Control

In agriculture and forestry it is well-known the existence of different microorganisms and insects that can cause production losses. Their monitoring and control are very important. In [33] a pest insect trap equipped with low-power image sensor technology is designed to perform remote automatic pest monitorization. Traps form a Wireless Sensor Network (WSN) and the images are sent via wireless one-hop broadcast communications to a host control station. In [34] is reported a system to detect the tobacco mosaic virus in the soil adopting the real-time quantitative polymerase chain reaction (RT-qPCR) by means of a specific device. A new technology for detecting root colonization in potatoes by microorganisms by exciting the material to produce fluorescence and capturing the images through a confocal laser scanning microscope is described in [35]. In [36] is outlined the design and realization of a bio-acoustic sensor, equipped with a probe for acquisition of sounds, in order to perform the early detection of real palm weevil for pest control.

## Machinery for Effective Treatments

In [37] is described the design and realization of a system composed by a 3D sonic anemometer and two axial fans able to perform the control and adjustment of sprayers in order to apply exactly the agrochemicals where they are required. In [38] is reported the detection of drift in vineyard spraying through a terrestrial Light Detection and Ranging (LIDAR) sensor. In [39] is reported the description of a system designed to estimate and predict the temperature and relative humidity during the tobacco drying process, using a dryer with a hot water valve to heat the air, a fan to move the air and two air hatchways to remove the humidity.

## Forest Stands and Reflectance

In [40] is described a system for forest stands identification for the production of mechanically-graded lumber based on the use of an acustic sensor. In [41] is reported the use of a terrestrial hyperspectral imaging spectrometer together with a laser scanner in order to obtain tree spectral reflectance characteristics. The incident light is measured by passing it through a line pattern diffraction grating to a mono-chromatic CCD.
